# Optimized High-Content Imaging Screening Quantifying Micronuclei Formation in Polymer-Treated HaCaT Keratinocytes

**DOI:** 10.3390/nano12244463

**Published:** 2022-12-15

**Authors:** Fariba Saadati, Walison Augusto da Silva Brito, Steffen Emmert, Sander Bekeschus

**Affiliations:** 1 ZIK *plasmatis*, Leibniz Institute for Plasma Science and Technology (INP), Felix-Hausdorff-Str. 2, 17489 Greifswald, Germany; 2 Clinic and Policlinic for Dermatology and Venereology, Rostock University Medical Center, Strempelstr. 13, 18057 Rostock, Germany; 3 Department of General Pathology, State University of Londrina, Rodovia Celso Garcia Cid, Londrina 86057970, Brazil

**Keywords:** algorithm, micro-plastic, nano-plastic, cytotoxicity, Operetta CLS

## Abstract

Research on nano- and micro-plastic particles (NMPPs) suggests their potential threat to human health. Some studies have even suggested genotoxic effects of NMPP exposure, such as micronuclei (MN) formation, while others found the opposite. To clarify the ability of NMPP to induce MN formation, we used non-malignant HaCaT keratinocytes and exposed these to a variety of polystyrene (PS) and poly methyl methacrylate (PMMA) particle types at different concentrations and three different sizes. Investigations were performed following acute (one day) and chronic exposure (five weeks) against cytotoxic (amino-modified NMPPs) and genotoxic (methyl methanesulfonate, MMS) positive controls. An optimized high-content imaging workflow was established strictly according to OECD guidelines for analysis. Algorithm-based object segmentation and MN identification led to computer-driven, unsupervised quantitative image analysis results on MN frequencies among the different conditions and thousands of cells per condition. This could only be realized using accutase, allowing for partial cell detachment for optimal identification of bi-nucleated cells. Cytotoxic amino-modified particles were not genotoxic; MMS was both. During acute and long-term studies, PS and PMMA particles were neither toxic nor increased MN formation, except for 1000 nm PS particles at the highest concentration of unphysiological 100 µg/mL. Interestingly, ROS formation was significantly decreased in this condition. Hence, most non-charged polymer particles were neither toxic nor genotoxic, while aminated particles were toxic but not genotoxic. Altogether, we present an optimized quantitative imaging workflow applied to a timely research question in environmental toxicity.

## 1. Introduction

Nano- and micro-plastic particles (NMPPs) are becoming a major environmental concern due to their small size, lack of biodegradability, and significant bio-permeability [1,2]. There is a continuous increase in the use of plastic in a variety of industries. A large proportion of plastic-containing products end up as waste in the environment due to the lack of efficient collection and improper management. In addition, many plastics do not enter the recycling chain [3]. These plastics are continuously degraded due to environmental stressors, such as salty sea water and UV radiation, and ultimately break down into smaller fragments to form NMPPs [4]. There are many ways through which humans can be exposed to NMPPs due to environmental contamination [5,6]. The skin is one of the primary human barriers against environmental pollution. Because it is the largest organ in the body, it can be a significant entry point for NMPPs into the human body. Skin and hair cosmetic products, such as scrubs, lotions, gels, and exfoliating products, are in contact with the various parts of the human body. A large-scale study of over seven thousand cosmetics and personal care products found 87% of them to contain micro-plastics [7]. Daily skin contact with micro-plastics can have destructive effects over time.

To date, only a few studies investigated whether plastic particles cause toxicity or genotoxicity in human skin cells. To reduce this shortcoming, the present study used human HaCaT keratinocytes to investigate genotoxic effects. The cell line is non-tumorigenic and capable of differentiation and regular keratinization as well as three-dimensional skin equivalent formation [8,9,10]. Accordingly, several types of toxicity analysis, e.g., silver nanoparticles [11] and cadmium [12], have been performed with this cell line, particularly on protection from DNA damage-inducing agents such as UV-radiation and oxidative stress [13,14,15]. Therefore, it seems plausible to have a well-established, non-tumorigenic, stress-sensitive skin cell line as a model system to study the biomedical consequences of plastic particle exposure, not only because the skin route is heavily understudied (despite the ample presence of NMPPs in cosmetic products and air pollution daily exposing the skin as the largest organ of the body to environmental particles) but also because of the suitability of the model system itself. At the same time, we aimed to provide a robust and donor-independent model system based on a commonly used cell line, which is more economical and reproducible in technical terms.

The literature on the genotoxic properties of NMPPs particles is not only still infrequent but also contradictory. Studies have shown both an increase in DNA damage when exposed to NMPPs and the absence of genotoxic damage [16,17,18]. The technical restrictions imposed on them can often lead to contradictory results, especially regarding the manual analysis of microscopy images. Therefore, we aimed to develop a new approach for evaluating genotoxicity based on the OECD-micronucleus protocol in vitro using high-content imaging and algorithm-driven quantitative image analysis. Micronuclei formation was chosen as the endpoint for genotoxicity because it detects an already-transmitted mutation [19]. Micronuclei are chromatin-containing bodies that appear due to DNA damage and may reflect a chromosome aberration. The in vitro micronucleus assay is a genotoxicity test system used to detect micronuclei in the cytoplasm of interphase cells. The micronuclei originate from chromosomes that cannot migrate to the poles during cell division [20]. It should be mentioned that the results of the in vitro studies published have investigated only short-time exposures to micro-plastic particles and, in most cases, high concentrations of micro-plastic particles [21].

Accordingly, this study aimed to investigate the toxic and genotoxic effects of several types (polystyrene and poly methyl methacrylate; PS and PMMA) and concentrations of polymer particles in human HaCaT keratinocytes in vitro. Besides acute effects, chronic effects were also investigated by analysing long-term polymer-HaCaT cultures for up to five weeks. We used a high-content imaging system and algorithm-driven quantitative image analysis to demonstrate these particles’ short- and long-term impact on HaCaT keratinocytes. Methyl methanesulfonate (MMS) was used as a positive control test chemical for micronuclei induction and method optimization, and amino-modified (NH_2_) PS particles with a diameter of 50 nm were used as positive particle control to monitor particle-induced toxicity. Accordingly, we comprehensively screened plastic particle exposure across different polymer types, particle sizes, and incubation times in HaCaT keratinocytes and in vitro for micronuclei formation using optimized quantitative high-content imaging and unsupervised algorithm-driven data analysis to infer the particles’ toxic and genotoxic effects.

## 2. Experimental Section

### 2.1. Cell Culture and Short-Term Polymer Treatment

HaCaT keratinocytes (Cell Line Service, CLS300493CP; passage number 40) were cultured in Roswell Park Memorial Institute (RPMI) medium 1640 containing 10% fetal bovine serum, 100 U/mL penicillin, and 100 µg/mL streptomycin. The cells were incubated at 37 °C and 5% CO_2_ in a humidified atmosphere and were passaged in a 1:3 ratio once per week when they reached approximately 80% confluency. Experiments were performed in 96-well plates containing a final volume of 100 μL of complete medium per well. For experiments, cells were seeded at an initial density of 1 × 10^3^ HaCaT keratinocytes per well. After incubation overnight to allow attachment, positive control dose–response analysis was performed by treating cells with various concentrations of PS-NH_2_ and MMS (methyl methanesulfonate) to find appropriate concentrations for toxicity and micronuclei induction. To achieve the optimum dose to score maximum bi-nucleated cells (BNCs) with less toxicity, cells were treated with cytochalasin B concentrations ranging from 0–9 µg/mL for 48 h. In the present study, 70–1100 nm PMMA and 50–1000 nm polystyrene particles were used. Particles were suspended in the PBS to set up the main stock solution. Following identifying dose–response data, the cells were treated with the desired dose of positive controls, 1 or 100 µg/mL concentrations of PS (50 nm, 200 nm,1000 nm, and mix of three sizes) or PMMA (70 nm, 400 nm, 1100 nm, and mix of three sizes) particles as a single treatment for 6 h. An exceedingly detailed analysis of the particles used in this study by dynamic light scattering for size, zeta-potential, and polydispersity, along with information on the manufacturer solvents, has been provided in a previous study [22]. The lower concentration of 1 µg/mL is a medium concentration that was claimed to be found in human blood of probands [23], although the methods of identifying this concentration are currently questioned in the community, and the results and concentration need to be verified in future studies. As complement, one higher (100 µg/mL) concentration was used. The cells were then treated with cytochalasin B at a final concentration of 2 µg/mL to block cytokinesis and allow the identification of MN in the cells that have completed one cell division and created BNCs.

### 2.2. Long-Term Polymer Treatment of HaCaT Keratinocytes

**Figure 1:**
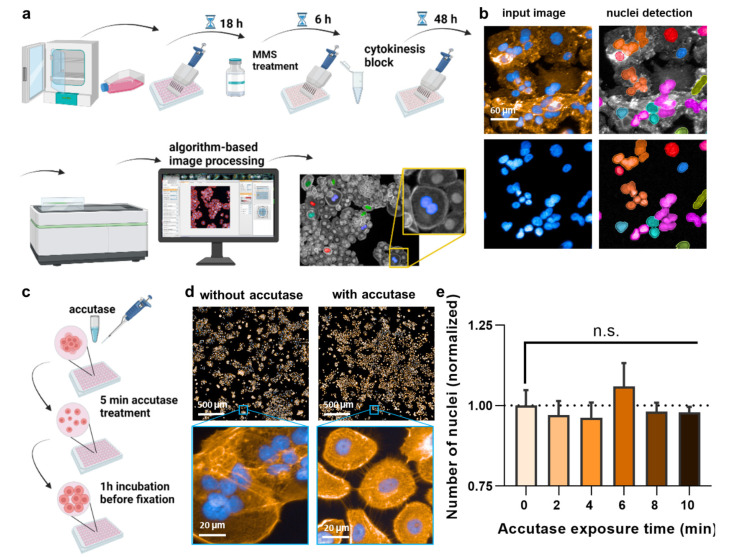
Study design and cell detachment. (**a**) Study design; (**b**) failure of original nuclear segmentation by software due to HaCaT keratinocytes’ aggregated cell growth; (**c**) scheme using a 5 min accutase treatment before fixation to lift and separate the cells without detachment; (**d**) representative image of the effect of accutase treatment on the separation of cells; (**e**) number of nuclei after 2–10 min of accutase exposure detachment, followed by addition of cell culture medium and 1 h incubation, and data normalization to cells not exposed to accutase (0), indicating that the initial accutase treatment does not lead to complete cellular detachment with subsequently less number of cells being identified by the software. Data are representative or mean + S.E.M. from at least three independent experiments. Statistical analysis was performed using one-way analysis of variances with Dunnett post hoc testing for multiple comparisons (**e**). n.s. = not significant.

To begin this process, 5 × 10^4^ HaCaT keratinocytes were suspended in complete cell culture media and seeded in T-25 flasks. Immediately after, the cell suspension was treated with 1 µg/mL NMPPs and incubated under optimum cell growth conditions. The culture medium containing NMPPs was replaced with culture medium containing NMPPs every two days to ensure continuous exposure. The cells were split every week. The experiments lasted approximately 5 weeks. This was to mimic constant exposure to plastic particles spanning approximately more than 30 cell division cycles to allow micronuclei to accumulate during each cellular (and nuclear) division. Considering a mean cell cycle time of 13 days in the epidermis [24], our long-term culture mimics more than 1 year of keratinocyte proliferation in vivo. Then, the cells of each group were detached from the flask and seeded separately in a 96-well plate. The next day, two different concentrations of NMPPs were added to the plate, similar to the acute treatment regime. Cell supernatants were replaced by fresh media containing 2 µg/mL cytochalasin B after 6 h (Figure 1a). The experiments were continued for two more days before imaging and MN assessment.

### 2.3. Staining and Imaging

The 96-well wells containing the cells were washed once with PBS and treated with 50 µL accutase for 10 min at room temperature. Accutase was deactivated by adding 50 µL of complete cell culture media, and the well plate was transferred to the incubator for 1 h. This additional incubation time helped a few residual cells that had become floating due to the accutase treatment to settle down to the well bottom again. The supernatant was then removed, and the cells were then fixed with 4% paraformaldehyde (PFA) for 15 min at room temperature, followed by washing twice with PBS. Cells were permeabilized by adding 0.1% Triton X-100 for 10 min. Non-specific binding was blocked by incubating cells with 2% bovine serum albumin in PBS for 30 min at room temperature. Alexa Fluor (AF) 594 phalloidin (BioLegend, Amsterdam, The Netherlands) working solution was prepared fresh by diluting 10 µL stock solution in 1 mL of PBS. Cellular actin was then stained using 50 µL of this working solution per well. Nuclei were then counterstained using a 2 µM working solution of DAPI at room temperature, followed by twice washing with PBS. Images were acquired using a high-content imaging system (Operetta CLS; PerkinElmer, Hamburg, Germany) in a single plane with a 20x air objective (NA = 0.4) to capture DAPI and AF594 fluorescence emission light. The non-confocal mode was used to acquire the images, and the binning was adjusted to 2. DAPI fluorescence was acquired with a λ_em_ 430–500 nm filter (λ_ex_ 365 nm), while a λ_em_ 570–650 nm filter (λ_ex_ 550 nm) was used to capture AF594 fluorescence. Per well and technical replicate and biological replicate, 61 fields of view were acquired. More than 12,000 individual fluorescence microscopy images were analyzed in this study. Algorithm-based image analysis was performed using *Harmony* 4.9 software (PerkinElmer).

### 2.4. Thiol Content

To begin this process, 1 × 10^5^ HaCaT keratinocytes were seeded in 24-well plates for 24 h, followed by exposure to either MMS (positive control) or NMPPs (at the previously described concentrations) for 6 h at 37 °C. Then, cells were washed with PBS afterward to remove excess NMPPs, and fresh culture medium containing cytochalasin B at 2 µg/mL was added for 48 h. Next, HaCaT intracellular thiol content was performed using a glutathione detection reagent (ThiolTracker Violet; Thermo Fisher Scientific, Bremen, Germany), and Zombie NIR (BioLegend) staining was used to mark dead cells. Cells were harvested and stained for 20 min at 37 °C. Single-cell acquisition was performed using flow cytometry (CytoFLEX LX; Beckman–Coulter, Krefeld, Germany). Flow cytometry data files were analyzed utilizing *Kaluza* 2.1.3 software (Beckman–Coulter).

### 2.5. Intracellular ROS

HaCaT keratinocytes (1 × 10^5^) were seeded in 96-well plates (Nunc; Thermo Fisher Scientific) at 37 °C for 24 h. Afterward, cells were exposed to MMS to either MMS (positive control) or NMPPs (at previously described concentrations) at 37 °C for 6 h. Cells were washed with PBS and stained with CM-H_2_DCF-DA (2 µM; Thermo Fisher Scientific) at 37 °C for 1 h. Images were acquired using a 20x objective and the Operetta CLS device described above. Excitation and emission settings were λ_ex_ 505nm and λ_em_ 510–570 nm for H_2_DCF-DA and digital phase contrast (DPC) was used for cells. Algorithm-based image quantification was performed using *Harmony* 4.9 imaging and analysis software (PerkinElmer). After flat field and bright field corrections, cells were detected by segmenting their cytoplasm using DPC signals. Then, the fluorescence intensities of cells were quantified, and the results were normalized to the total cell area analyzed. The final results were expressed as mean fluorescence intensity (MFI) per cell area and were normalized against vehicle controls.

### 2.6. Statistical Data Analysis

Statistical analysis was performed using one-way analysis of variances with Dunnett’s post hoc test to correct for multiple testing or *t*-test. Levels of significance were indicated as follows: α = 0.05 (*), α = 0.01 (**), α = 0.001 (***). Graphing and statistical analysis were performed using *prism* software 9.4.1 (GraphPad; San Diego, CA, USA).

## 3. Results

### 3.1. Method Optimization of MN Formation Analysis in HaCaT Keratinocytes

**Figure 2:**
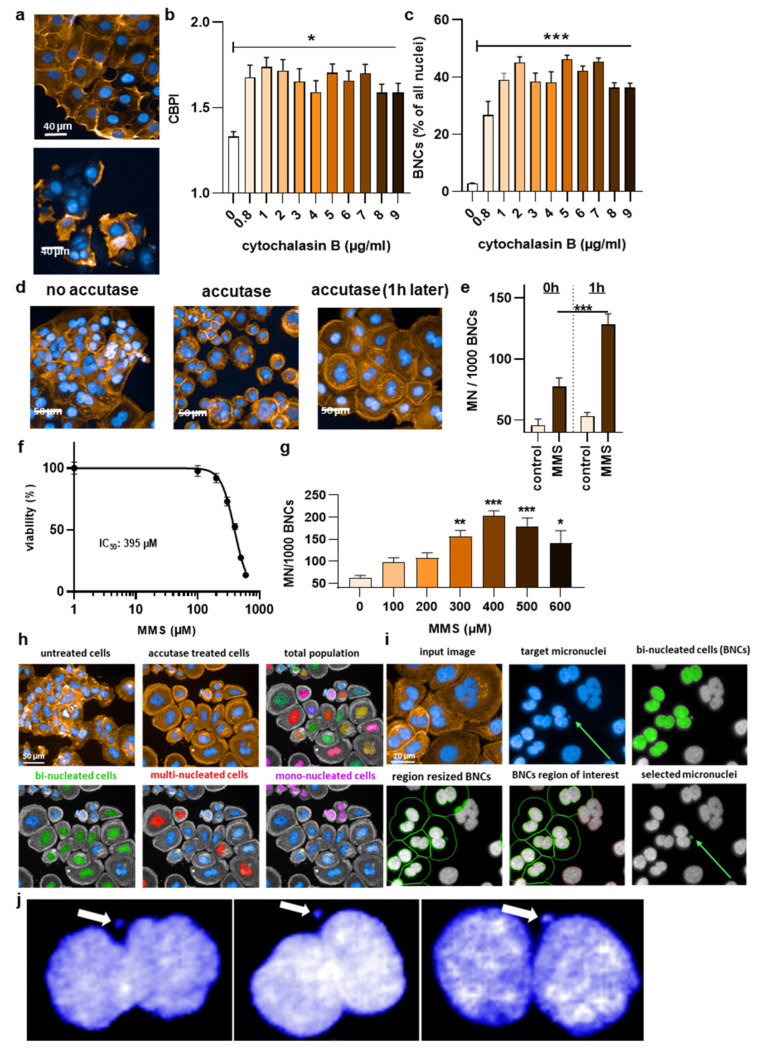
High-content imaging protocol optimization. (**a**) Representative images for the effects of low (upper image) and high (lower image) cytochalasin B concentrations; (**b**) cytokinesis block proliferation index (CBPI) in relation to different cytochalasin concentrations; and (**c**) bi-nucleation rate in relation to different cytochalasin concentrations; (**d**) representative images of the effect of accutase on nuclear segmentation and single cells; (**e**) micronucleation rate at 0 h and 1 h post accutase treatment; (**f**) MMS dose–response curve; (**g**) MMS concentrations in relation to micronucleation rate among bi-nucleated cells (BNCs); (**h**) representative images showing the benefit of accutase treatment to identify nuclei, and algorithm-based sub-grouping from the total population into bi-nucleated cells (BNCs), multinucleated cells, and mono-nucleated cells; (**i**) representative images from the algorithm-driven object segmentation workflow starting with the input image and the identification of bi-nucleated cells from the DAPI-stained nuclear over the identification of the cytosolic area via the phalloidin-stained actin (orange), the removal of cross-contaminating nuclei in these image regions, and the automatic identification of micronuclei (MN) related to bi-nucleated objects; (**j**) representative magnified fluorescence microscopy images of DAPI staining in bi-nucleated cells with micronuclei. Data are representative or mean +/± S.E.M. from at least three independent experiments. Statistical analysis was performed using one-way analysis of variances with Dunnett post hoc testing for multiple comparisons (**b**,**c**,**g**) or *t*-test (**e**) with * = *p* < 0.05, ** = *p* < 0.01, and *** = *p* < 0.001.

To date, a reliable method to analyze micronuclei (MN) formation with an algorithm-based method is not available for HaCaT keratinocytes. This is because of the clustered growth of the cells, impeding image-segmentation-based detection of individual cells and nuclei. This, again, is important because, according to the OECD protocol No. 487, the MN must only be counted in bi-nucleated cells (BNCs) and ultimately related to the absolute numbers of the latter. Quantitative fluorescence analysis has the advantage of identifying hundreds to thousands BNCs relatively quickly, out-competing manual counting attempts in terms of number, accuracy, and unbiasedness. Accordingly, a cytokinesis-block micronucleus assay was set up (Figure 1a). In regularly fixed and stained cells (blue: nuclei; orange: actin/cytosol), neither unambiguous identifications of BNCs nor their cytosolic region could be identified (Figure 1b). Strikingly, the clustered growth can be alleviated by incubating the cells with accutase (Figure 1c) long enough to become single cells in the 96-well plate (Figure 1d) but too short for the cells to detach completely. Quantifying the absolute number of nuclei (and normalization to control-wells corresponding to “0 min” that had not received accutase) showed no difference for incubation times up to 10 min with accutase (Figure 1e), indicating the accutase effect only separated but not completely detached the cells. Next, cytochalasin B was used, an agent shown to prevent metastasis [25] and cytotoxic at higher concentrations (Figure 2a). It is added to cells to inhibit actin polymerization, thereby inhibiting cytokinesis and allowing the identification of MN in cells that had undergone one cell and nuclear division but not separation. Cells were sampled after 48 h, a time point equivalent to about 1.5–2.0 normal cell cycle lengths. The cytokinesis-block proliferation index (Figure 2b) and the percentage of BNCs (Figure 2c) were significantly increased for low to modest cytochalasin B concentrations used. The ideal dosage of cytochalasin B was determined to be 2 µg/mL since it was not highly toxic to HaCaT keratinocytes and allowed us to obtain 50% bi-nucleated cells at 48 h reliably. Next, the incubation time post-accutase treatment was optimized (Figure 2d), and a manual quantification of MN revealed 1 h incubation to be significantly superior to 0 h (Figure 2e). Next, the dose–response of methyl methanesulfonate (MMS) treatment, a genotoxic compound and positive control in this study [26], was analyzed (Figure 2f). The number of manually quantified MN reached a maximum at about 400 µM (Figure 2g), but as this concentration already was toxic in 50% of cells, 300 µM was used for further analysis. The final algorithm-driven image analysis could now be set up. Representative images of the object segmentation workflow are provided (Figure 2h), showing the benefit of accutase treatment to identify nuclei, and algorithm-based sub-grouping from the total population into bi-nucleated cells (BNCs), multinucleated cells, and mono-nucleated cells. From there, bi-nucleated cells (BNCs) were identified from the DAPI-stained nuclear over the identification of the cytosolic area via the phalloidin-stained actin (orange), the removal of cross-contaminating nuclei in these image regions, and the automatic identification of micronuclei (MN) related to bi-nucleated objects (Figure 2i) as highlighted (Figure 2j).

### 3.2. Acute PS and PMMA Polymer Particle Toxicity and Genotoxicity Were Negligible

**Figure 3:**
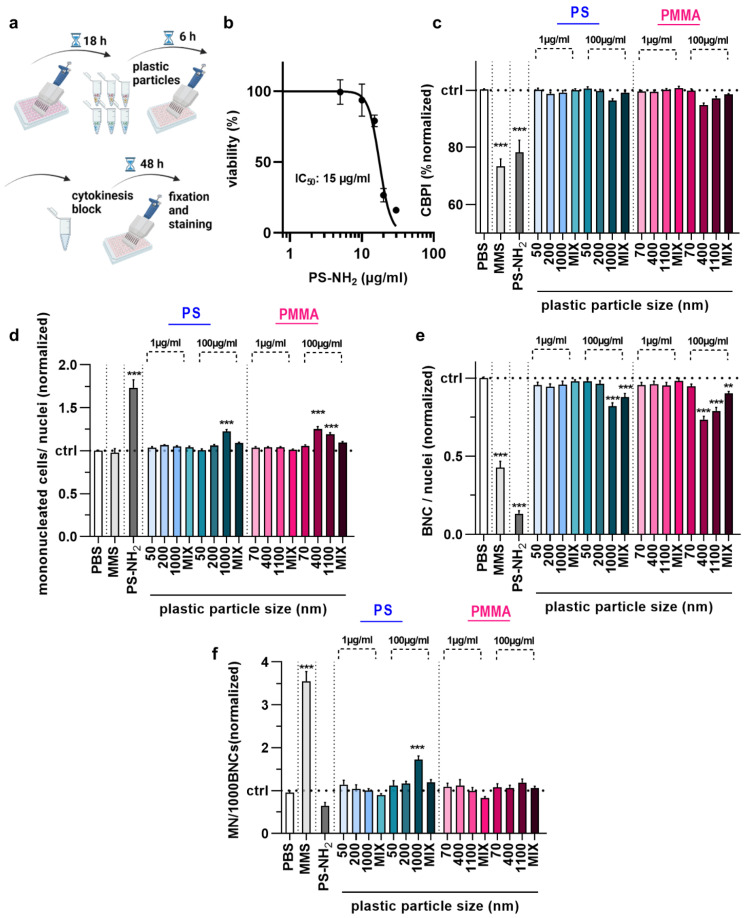
Acute toxicity and genotoxicity testing in HaCaT keratinocytes upon polymer particle exposure. (**a**) Acute particles exposure scheme; (**b**) aminated (PS-NH_2_) polystyrene (PS) particle dose–response curve 54 h post-exposure; (**c**–**f**) CBPI (**c**), number of MNCs (**d**), number of BNCs (**e**), and MN frequency per 1000 BNCs (**f**) in HaCaT keratinocyte samples exposed to vehicle (PBS), MMS (positive control for genotoxicity), PS-NH_2_ particles (positive control for polymer toxicity), and PS and PMMA polymer particles of different sizes (50–1100 nm) and concentrations (1 µg/mL and 100 µg/mL). Data are representative or mean + SEM from at least six independent experiments. Statistical analysis was performed using one-way analysis of variances with Dunnett post hoc testing for multiple comparisons (**c**,**d**,**e**,**f**) with ** = *p* < 0.01 and *** = *p* < 0.001.

**Figure 4:**
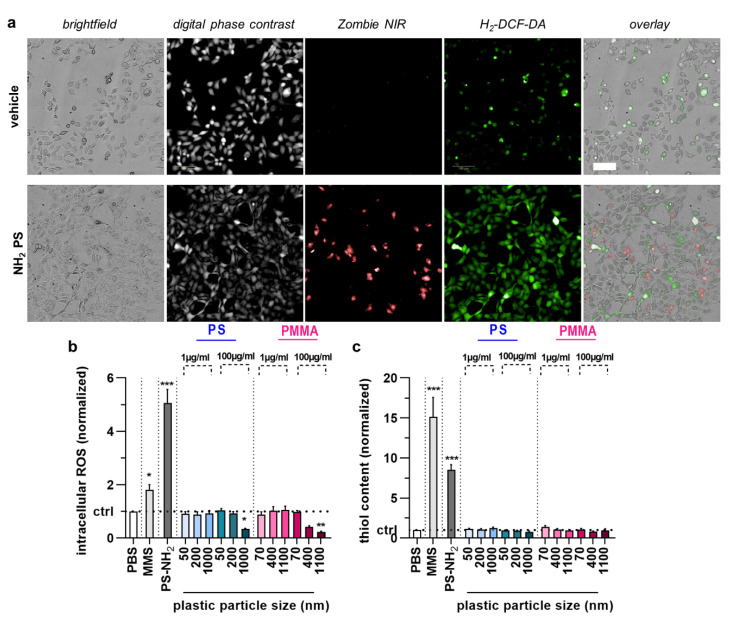
Cell cycle arrest, intracellular ROS, and antioxidant defense in HaCaT keratinocytes upon acute polymer particle exposure. (**a**) Representative brightfield, digital phase contrast, Zombie NIR, H2-DCF-DA, and overlay images of vehicle and NH_2_ particle treated HaCaT keratinocytes; (**b**,**c**) intracellular ROS (normalized DCF mean fluorescence intensity) (**c**), and antioxidant defense (normalized thioltracker violet mean fluorescence intensity) (**c**) in HaCaT keratinocyte samples exposed to vehicle (PBS), MMS (positive control for genotoxicity), PS-NH_2_ particles (positive control for polymer toxicity), and PS and PMMA polymer particles of different sizes (50–1100 nm) and concentrations (1 µg/mL and 100 µg/mL). Data are representative or mean + SEM from at least four independent experiments. Statistical analysis was performed using one-way analysis of variances with Dunnett post hoc testing for multiple comparisons with * = *p* < 0.05, ** = *p* < 0.01, and *** = *p* < 0.001.

We next applied the newly established method to analyze the acute toxicity and genotoxicity of two types of polymer particles (PS and PMMA) across three sizes each (50 nm and 70 nm, 200 nm and 400 nm, 1000 nm and 1100 nm, respectively) as well as one size mix for two different concentrations (1 µg/mL and 100 µg/mL) in HaCaT keratinocytes in vitro. The cells were incubated with particles for 6 h, followed by performing the CBMN assay as described above (Figure 3a). As positive control, aminated (NH_2_) particles (size: 50 nm) were used, and their IC_50_ was determined (Figure 3b); 10 µg/mL were used for subsequent experiments. We then calculated the CBPI of cells. The results indicate lower proliferation in cells treated with MMS or aminated particles, as both substances are cytotoxic (Figure 3c). By contrast, none of the particle exposure conditions affected the calculated CBPI values of HaCaT cells significantly. This, however, was not the case for the number of mononucleated (Figure 3d) and bi-nucleated cells identified (Figure 3e) as well as the number of micronuclei among BNCs (Figure 3f). Specifically, the larger particle sizes at the high concentrations elevated MNC and BNC counts. At the same time, only 1 µm PS at 100 µg/mL led to a modest but significant increase in the MN frequency. It has been suggested that increased MN frequencies are associated with oxidative stress. To this end, we tested intracellular ROS production in HaCaT keratinocytes exposed to polymer particles (Figure 4a). ROS production was elevated for positive controls. In contrast, intracellular ROS levels were significantly decreased for 1000 nm PS and 1100 nm PMMA particles at the high 100 µg/mL concentration (Figure 4b). As this was surprising, we analyzed whether this ROS decrease affected the cells’ antioxidative defense capacity. By quantifying intracellular free thiols, we identified profound changes with the positive control treatment of HaCaT keratinocytes, while none of the polymer particle conditions led to significant alterations in the antioxidant defense capacity (Figure 4c).

### 3.3. Chronic PS and PMMA Polymer Particle Toxicity and Genotoxicity Were Similar to Acute Toxicity In Vitro

**Figure 5:**
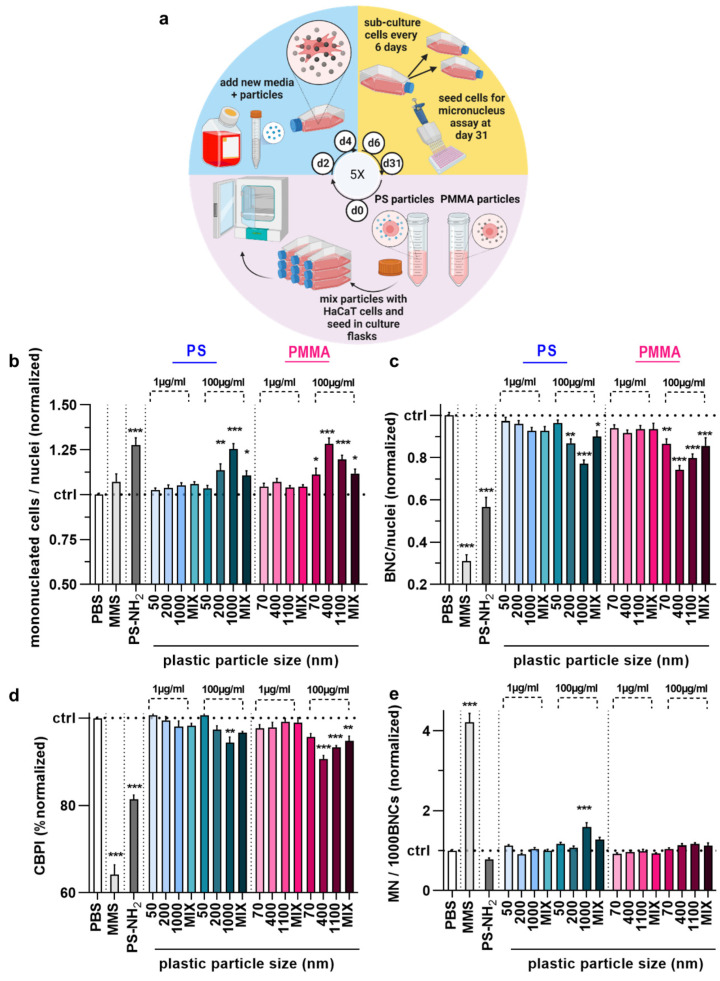
Long-term (5-week) genotoxicity testing in HaCaT keratinocytes upon polymer particle exposure. (**a**) study scheme; (**b**–**e**) mononucleated (**b**), bi-nucleated (**c**), CBPI (**d**), and micro-nucleated (MN) € cells per 1000 BNCs of HaCaT keratinocytes exposed to vehicle (PBS), MMS (positive control for genotoxicity), PS-NH_2_ particles (positive control for polymer toxicity), and PS and PMMA polymer particles of different sizes (50–1100 nm) and concentrations (1 µg/mL and 100 µg/mL) for 6 h post chronic exposure to the respective particles for 5 weeks at 1 µg/mL. Data are representative or mean +SEM from at least six independent experiments. Statistical analysis was performed using one-way analysis of variances with Dunnett post hoc testing for multiple comparisons (**b**–**e**) with * = *p* < 0.05, ** = *p* < 0.01, and *** = *p* < 0.001.

The majority of studies investigating the effects of plastic exposure in vitro focus on short-term investigations reflecting acute consequences. By contrast, we also wanted to elucidate the long-term effects of plastic exposure by setting up a 5-week permanent plastic particle culture model (Figure 5a). Before conducting the MN analysis of the cells permanently cultured in the presence of NMPPs (chronic exposure) at a final concentration of 1 µg/mL, the cells were exposed to the CBMN assay protocol exactly as described for the short-term (acute) exposure. Overall, the results obtained for mononucleated (Figure 5b) and bi-nucleated (Figure 5c) HaCaT keratinocytes were similar to those of acute exposure. Specifically, larger particles at higher concentrations of 100 µg/mL led to elevated MNC and decreased BNC numbers in HaCaT keratinocytes. Additionally, the CBPI (Figure 5d) was, in contrast to short-term exposure, significantly decreased for the cells incubated in the presence of particles of larger sizes and at higher concentrations. Regarding the MN frequency, the same 1 µm PS culture conditions at 100 µg/mL gave a low but significant increase in MN frequency (Figure 5e), similar to findings with acute exposure (Figure 3f). All other plastic particle culture conditions did not induce changes in HaCaT keratinocytes. Collectively, our data suggest polymer particles of larger sizes (>1000 nm) to have greater effects on cell cultures in vitro.

## 4. Discussion

The exponential growth of plastic waste has been observed worldwide, which causes environmental contamination [27]. However, the reports on the effects of nano- and micro-plastic particles (NMPPs) on human health are conflicting, ranging from little to more pronounced effects. Yet, even though several studies demonstrate that NMPPs may accumulate in cells without producing acute toxicity, the fact that certain NMPPs have been found to impact cells is a reason for concern [28,29,30,31]. This confirms that their interactions with biological systems should be investigated for the potential to cause toxicity and genotoxicity. We attempted to investigate this thoroughly across several iterations on polymer type, size, concentration, acute or chronic exposure, and by using algorithm-driven quantitative imaging.

NMPPs can enter cells and interact with them using different pathways. The uptake of NMPPs was evaluated and proved in our previous study in three different cell lines [22]. Here, we aimed to explore the genotoxic effects of PS and PMMA particles in human epidermal keratinocytes. The high-content imaging-based method and software were established to identify and quantify micronucleus formation in an unprecedented fashion. The most crucial factor while performing an MN assay based on OECD guidelines is verifying that the cells being scored have completed mitosis throughout the treatment or post-treatment period [32]. Cytochalasin B is a substance that has been frequently used to block cytokinesis. It blocks actin assembly during cytokinesis and prevents daughter cell separation after mitosis, resulting in bi-nucleated cell formation [33,34,35]. Humans can be exposed to NMPPs for a long period at low concentrations. Notwithstanding, OECD guidelines on the micronucleus assay suggest that the treatment period with toxicants should be short in cytogenetic assays [36]. Accordingly, higher concentrations of toxicants should be preferred for exposure to detect subsequent genetic damage. In this study, we tested low (1 µg/mL) and high (100 µg/mL) concentrations of NMPPs in in vitro conditions. NMPPs at high concentrations may produce biologically irrelevant positive results. However, based on the results obtained from relatively higher concentrations of NMPPs, influential information can be deduced from evidence obtained for lower concentrations for a long period of exposure [37,38]. In our previous study, we monitored the uptake and intracellular localization of NMPPs and found that 6 h was enough for particles to enter the cells despite varying uptake kinetics for various cell lines [22].

The NMPPs’ potential ability to induce genotoxicity can be divided into direct and indirect damage. As the direct mechanism of genotoxicity, the damage can occur through the direct physical interaction of NMPPs with the DNA of a cell, and the effect of indirect genotoxicity can be induced through oxidative stress causing damage to proteins involved in the replication of DNA and the division of cells [29,39]. Hence, investigating ROS could be a fundamental point in determining the underlying mechanism involved in the toxicity and genotoxicity of NMPPs. Therefore, we evaluated ROS production induced by NMPPs exposure and analyzed and quantified cellular thiol content. PS-NH_2_ particles have been used here as a positive control to illustrate particles’ toxic, especially ROS-provoking effect, given their impact on cell proliferation [40]. Surprisingly, despite the toxic effect, the number of MN/1000 BNCs was lower in PS-NH_2_-treated cells compared to untreated cells. Additionally, mononucleated cell numbers were significantly higher in the cells treated with these particles. Comparing the number of mononucleated cells and BNCs in PS-NH_2_ treated cells explained how these particles might potentially interfere with DNA replication and cell division [41,42]. Hence, the data suggest that the treatment with PS-NH_2_ interfered with the cell cycle progression and inhibited DNA synthesis in HaCaT keratinocytes [42]. In A549 lung cancer cells, Kim and colleagues reported that PS-NH_2_ induced cell death and interrupted cell cycle progression, and cell cycle arrest occurred gradually at two points along the cell cycle [41]. Lunov and colleagues previously investigated the effect of PS-NH_2_ on macrophages differentiated from human monocyte and reported that these particles potentially led to cell death and inflammation [43]. However, additional experiments are needed to confirm this point and further elucidate cell cycle arrest mechanisms.

Interestingly, PS particles similar to the PS-NH_2_ but without surface modification in three different sizes do not show any cytotoxic effect over two concentrations in acute treatment. However, the rate of BNCs per nuclei suggests that a high dose of NMPPs can induce cell cycle arrest, depending on the exposure dose. The number of mononucleated cells confirms these results, which are also in line with other studies [44,45]. Genotoxicity was another crucial toxicological endpoint that was evaluated. Our study did not find any relevant genotoxic effect in the cells exposed to NMPPs for 6 h, except a small but significant increase for one 1 µm PS particle condition at 100 µg/mL. For all other conditions, we observed that the treatment of the NMPP’s low concentration did not change the frequencies of MN as a genomic instability in the HaCaT keratinocytes compared to the background. Although in some studies, higher levels of DNA damage have been recorded in cells treated with NMPPs, others have found no correlation between NMPP exposure and genotoxic damage [17,39,46], confirming our findings. The same is true for the absence of major cytotoxic effects for particles of different types, origins, sizes, and concentrations [47,48,49]. Yet, cell membrane alterations could result from the internalization of the NMPP and direct physical contact between the membrane and NMPPs. Bigger particles have more contact with the cells’ surface. It has been suggested that, especially during prolonged exposure of cells to NMPPs in vitro, the particles interact continuously with the cells and elicit various changes in the cell membrane [50,51,52,53]. As a result, cells may be more vulnerable to high particle dosage, potentially leading to cell cycle arrest and cell death after prolonged exposure. This explains why statistically significant results were not found at the lower concentrations, while significant changes were observed at 100 μg/mL concentrations after prolonged exposure in our study. Similar to acute treatment, the BNCs rate showed that NMPPs treatment caused cell cycle arrest, and cells could not pass the cell division process. As a result, there are more mononucleated cells and fewer BNCs [50,54,55,56,57]. Regarding NMPP size, we investigated not only individual sizes but also the effects of NMPP mixtures of different sizes. Interestingly, the results of this “mix” treatment in HaCaT cultures permanently incubated in the presence of plastic particles resembled the effects seen with the smaller than larger particles (Figure 5). The authors were not fully prepared for the result of exposing the cells to a mixture of plastic particles of various sizes. Hence, there is no acceptable explanation for these findings.

Several studies reported that, following NMPPs uptake by cells, they aggregate in lysosomes, causing lysosomal swelling and membrane damage, which releases proteolytic enzymes into the cytosol and triggers cell death [50,58]. Along those lines, it has also been suggested that NMPPs exposure is associated with increased oxidative stress. However, since some studies have reported direct toxicity without ROS generation, the assumption that ROS generation is necessary for NMPPs-induced toxicity and genotoxicity may be incorrect. The results of our study corroborate these findings. However, to predict NMPP-induced toxicity, ROS generation in cells exposed to NMPPs must be characterized to determine whether ROS generation plays a role in NMPP-induced harmful effects [30,59,60]. In addition, various potential molecular mechanisms might be responsible for creating MN in response to the presence of NMPPs. Micronuclei may be clastogens, chromosome fragments created when DNA is broken, or aneugens, complete chromosomes created when the mitotic apparatus is disrupted. NMPPs seem to be somewhat in between, suggesting they are both clastogenic and aneugenic. However, more details on the mechanisms will be investigated in future studies on this subject.

The limitation of this study was that the results on NMPP genotoxicity are only applicable to in vitro HaCaT keratinocytes cultures, and we do not extrapolate our findings to draw conclusions on the limited genotoxicity of NMPPs in human skin. From a dermatological point of view, testing primary keratinocytes and using 3D skin models would be desirable to obtain results in model systems that more closely mimic in vivo conditions. In addition, the chronic exposure cultures could have been performed for longer, and extending the assay toward other cell lines is desirable. Moreover, we only tested homogenous, commercially available plastic particles, and it would be interesting to test environmental nano- and micro-plastic samples in future studies.

## Data Availability

Data from this manuscript are available upon request.
